# The effect of adrenocorticotropic hormone on alpha‐2‐macroglobulin in osteoblasts derived from human mesenchymal stem cells

**DOI:** 10.1111/jcmm.15152

**Published:** 2020-03-12

**Authors:** Faezeh Sadeghi, Elham Vahednia, Hojjat Naderi Meshkin, Mohammad Amin Kerachian

**Affiliations:** ^1^ Medical Genetics Research Center Mashhad University of Medical Sciences Mashhad Iran; ^2^ Department of Medical Genetics Faculty of Medicine Mashhad University of Medical Sciences Mashhad Iran; ^3^ Department of Molecular Genetics Islamic Azad University Damghan Iran; ^4^ Stem Cell and Regenerative Medicine Research Department Mashhad Branch Academic Center for Education, Culture and Research (ACECR) Mashhad Iran

**Keywords:** adrenocorticotropic hormone, alpha‐2‐macroglobulin, avascular necrosis, mesenchymal stem cells, osteoblasts

## Abstract

Nowadays, alpha‐2‐macroglobulin (*A2M)* gene has allocated escalating interest among several genes involved in the pathogenesis of avascular necrosis of the femoral head (ANFH). This molecule could interact with several osteogenic‐related proteins. It was reported that adrenocorticotropic hormone (ACTH) affects bones through its receptor located on osteoblasts, suggesting it as a potential target in ANFH treatment. In this study, the effect of ACTH on *A2M* expression was investigated in osteoblasts as well as during the differentiation of human mesenchymal stem cells (MSCs) into osteoblasts. In this study, MSCs derived from bone marrow were isolated and purified using Ficoll gradient and several passaging. MSCs were characterized by induction with osteogenic and adipogenic medium followed by Oil Red O, Alizarin Red and alkaline phosphatase staining. Besides, MSCs were exposed to various concentrations of ACTH to evaluate the cell variability by MTT assay. MSCs and differentiated osteoblasts were treated with 10^−8^ molar ACTH for 16 and 26 days, respectively. Then, the total RNA was extracted and *A2M* expression was quantified by real‐time qPCR. The protein expression levels of osteoblast markers including alkaline phosphatase (*ALPL*) and bone gamma‐carboxyglutamate protein (*BGLAP*) were also measured. The results showed that *A2M* expression in cells treated with ACTH was up‐regulated significantly compared to the control group. Similarly, the expression of osteoblast gene markers including *ALPL* and *BGLAP* was significantly increased. ACTH, as an osteoblastic differentiation enhancer, up‐regulates *A2M*, which promotes osteoblastic differentiation probably through *TGF‐β* induction.

## 1 INTRODUCTION

Avascular necrosis of femoral head (ANFH) is a pathologic process, which leads to hip joint destruction as well as bone loss in femoral head.^1^ It results from interruption of blood supply to the epiphyses of the femur.^2^ Nearly, 50% of patients with ANFH require a major surgical procedure, for example total hip replacement (THR) for treatment.^3^ Although therapies such as THR are an available procedure for controlling this problem,^4^ it has its own disadvantages such as high morbidity rate. Besides, the patients have to suffer from additional major operations later in their lives.^4^, ^5^ Thus, finding novel diagnostic methods especially in early stages of the disease and modern therapeutic approaches including stem cell therapy are strongly required.^6^

Mesenchymal stem cells (MSCs) have allocated a lot of interest because of their potency to repair and regenerate musculoskeletal tissues. MSCs can be easily isolated from different tissues but the main source is bone marrow called the human bone marrow mesenchymal stem cells (hBM‐MSCs). These multipotent cells can differentiate into various lineages such as osteoblasts, chondrocytes and adipocytes.^7^ Differentiation of osteoblasts and chondrocytes can be influenced by various substances such as adrenocorticotropic hormone (ACTH), a melanocortin peptide hormone derived from proopiomelanocortin (POMC).^8^ ACTH directly increases bone markers related to expression of adrenocorticotropic receptor on the surface of MSCs and osteoblasts derived from MSCs.^9^ Furthermore, it was reported that this hormone has a protective role against ANFH.^6^ Thus, it could be used as a potential therapeutic agent in ANFH treatment. Its therapeutic response probably arises from an elevated osteoblastic support and the stimulation of VEGF by ACTH; the latter is largely responsible for vascularization, which surrounds highly remodelling bone.^6^

Previously, a significant overexpression of alpha‐2‐macroglobulin gene (*A2M*) in a glucocorticoid‐induced rat model of ANFH has been reported,^10^ and in a recent study, it has been shown that the serum protein level of this protein interestingly raised in ANFH patients.^11^ This protein has several roles in fibrinolysis and the coagulation cascade.^12^ Besides, it is a carrier of diverse growth factors and cytokines contributing to osteogenesis.^13^, ^14^, ^15^, ^16^ It is feasible that ACTH influences the osteogenesis and vascularization via influencing the gene expression of A2M as a role player in ANFH. In the present article, we investigated the effects of ACTH on *A2M* expression in osteoblasts derived from hBM‐MSCs and in the process of their differentiation. This probably could assist to have a better understanding of the pathogenesis of ANTH, discovering more effective therapeutic strategies.

## 2 MATERIALS AND METHODS

### 2.1 Human MSCs isolation and expansion

The bone marrow aspirates were taken from femur of normal adult donors after informed consents were signed by the participants and under the protocol approved by the research ethics committee of Mashhad University of Medical Sciences (MUMS) with the ethical code of 922645. Mononuclear cells were isolated by Ficoll‐Paque PLUS (GE Healthcare) and density gradient centrifugation; then, they were plated in tissue culture flasks in Dulbecco's modified Eagle's medium (Invitrogen) with 10% foetal bovine serum (Gibco) supplemented with 2 ng/mL basic fibroblast growth factor (Royan Institute) and 100 U/mL penicillin‐streptomycin (Gibco). In the following, the cells were incubated at 37°C in a humidified atmosphere with 5% CO_2_. After cell incubation for 3 days, culture medium was refreshed to remove non‐adherent cells. Then, adherent cells were cultured until they reached 70%‐80% confluency. After that, the cells were detached by trypsinization with 0.25% trypsin‐EDTA (Gibco) followed by subculturing to new flasks. Finally, MSCs in passage 4 were used in our experiments.

### 2.2 MSCs characterization

Osteogenic and adipogenic differentiation was performed in order to check MSCs characteristics. Briefly, 2 × 10^4^ cells/well were seeded in 6‐well plates and the medium was changed every other day with differentiation medium.

### 2.3 In vitro osteogenic differentiation

To induce osteogenic differentiation, MSCs in passages 4 were cultured under osteogenic conditions containing expansion medium supplemented with 100 nmol/L dexamethasone sodium phosphate (DarouPakhsh), 0.2 mmol/L L‐ascorbic acid 2‐phosphate (Sigma) and 10 mmol/L β‐glycerol phosphate (Sigma‐Aldrich). After 21 days of incubation, the differentiated cells were stained with Alizarin Red S (Sigma‐Aldrich) and 5‐bromo‐4‐chloro‐3‐indolyl phosphate/nitro blue tetrazolium (BCIP/NBT) substrate (Sigma‐Aldrich).

### 2.4 In vitro adipogenic differentiation

The cells in passage 4 were cultured under adipogenic conditions for 16 days in order to induce adipogenic differentiation. Adipogenic medium consists of expansion medium supplemented with 100 nmol/L dexamethasone sodium phosphate and 100 µmol/L indomethacin (Sigma‐Aldrich). After 16 days, cells were stained with Oil Red O (Sigma‐Aldrich).

### 2.5 Alizarin Red S staining

Alizarin Red staining was performed to detect matrix mineralization. Following hBM‐MSCs culturing in osteogenic medium for up to 16 days, the cells were fixed with 4% paraformaldehyde for 30 minutes. Then, the fixed cells were stained with 1% Alizarin Red S, pH = 4.1‐4.3 (Sigma‐Aldrich, Germany) for 45 minutes. After that, cells were washed with ddH_2_O to remove excess stain. Finally, mineralization was checked by light invert microscopy and photographed.

### 2.6 Alkaline phosphatase staining

After culture of hBM‐MSCs in dedicated time, cells were fixed with 4% paraformaldehyde and washed with phosphate buffered saline (PBS). Then, the cells were incubated with BCIP/NBT (5‐bromo‐4‐chloro‐3‐indolyl phosphate/nitroblue tetrazolium liquid substrate) (Sigma‐Aldrich, Bornem, Belgium) for 10 minutes. Treated cells were washed with ddH_2_O and examined with invert microscopy. Positive staining was visualized as dark purple colour.

### 2.7 Oil Red O staining

Induced cells were rinsed with PBS and fixed with 4% paraformaldehyde for 30 minutes. After washing with ddH_2_O, cells were coated with 60% isopropanol. Then, cells were incubated with Oil Red O working solution [diluted stock solution with water (3:2)] for about 15 minutes. To intensify the staining of the nuclei, haematoxylin was used. In the final step, the lipid droplets were checked microscopically as red colour with purple nucleus.

### 2.8 Flow cytometric analysis

MSCs in passage 4 were grown until 100% confluency and characterized with flow cytometry for expression of CD105, CD44, CD90, CD34, CD11b and CD45 surface markers (Exbio, Prague, Czech Republic). Briefly, the cells have been trypsinized and precipitated by centrifugation at 600 g for 4 minutes. About 2 × 10^5^ cells were incubated with each antibody for 1 hour at 4 ⁰C, separately. Then, the cells were centrifuged at 600 *g* for 4 minutes. The supernatant was removed, and the participate was resuspended in 100‐200 µL washing buffer containing 2%‐5% FBS in PBS. The surface expression of mentioned markers was evaluated using flow cytometer (BD FACSCalibur). Results were analysed using Flowing Software version 2.2.

### 2.9 Preparation of various concentrations of ACTH

To prepare 9 different concentrations of ACTH (10^−4^ to 10^−12^ molar), 0.0145 gr of ACTH was dissolved in 500 µL sodium chloride (stock concentration: 6.4 mmol/L). Required concentrations of ACTH were prepared by diluting different volumes of this solution in proper amounts of culture medium.

### 2.10 MTT assay

MTT [3‐(4, 5‐dimethylthiazol‐2‐yl)‐2, 5‐diphenyltetrazolium bromide] assay was performed to investigate the cytotoxic effects of ACTH. MTT test is dependent on cleaving the tetrazolium salt and forming purple formazan crystals.^17^ To do so, cells were trypsinized after reaching 80% confluency and resuspended in culture medium. Then, 5 × 10^3^ cells were plated in 96‐well plates (JET BIO) and treated with various concentrations of ACTH for three consequent days. To evaluate viability of treated cells, MTT solution was prepared freshly by dissolving 5 mg MTT dye (Sigma) in 1 mL PBS and filtered through 0.2‐μm filter (JET BIO). After that, 20 μL of MTT was added to each well followed by incubation at 37°C for 4 hours. Then, MTT solution was removed and dimethyl sulfoxide (DMSO; 200 μL/well) was used to dissolve purple formazan. Last, optical density (OD) was measured by multiwell scanning spectrophotometer (ELISA reader, Awareness, USA) at 495 nm. For calculating living cells (%), the OD of treated cells/well was divided into mean OD of control cells × 100.

### 2.11 ACTH treatment

MSCs were treated with differentiation medium and supplemented with 10^−8^ molar ACTH for 16 days. Besides, in another group, MSCs‐differentiated osteoblasts were exposed to the same concentration of ACTH for an extra 10 days.

### 2.12 RNA preparation and gene expression analysis

Total RNA was extracted using AccuZol™ reagent (Bioneer) followed by DNase 1 (Qiagen) treatment. Briefly, 1 mL of AccuZol™ was added per 10 cm^2^ area of the culture dish. Then, 200 µL of chloroform (Merck) was added per 1 mL AccuZol™, and the samples were centrifuged at 16 000 *g* for 15 minutes. In the following step, the aqueous phase was transformed to a new microtube and the equal volume of isopropanol (Merck) was added to each tube and centrifuged at 16 000 *g* for 10 minutes. After removing the supernatant, 1 mL of ethanol (Merck) was added to the microtube and centrifuged at 16 000 *g* for 5 minutes. The pellet was dissolved in RNase‐free water (ParsTous) and stored at −70°C.

Total RNA (1 µg) was used to first‐strand cDNA synthesize by PCR, using reverse transcriptase (Fermentas). Transcript levels were quantified by SYBR^®^ Green Real‐Time PCR Master Mix (ParsTous). Real‐time qPCR assay was done in triplicates in a 20 µL reaction buffer consisting of 10 µL 2× SybrGreen master mix, 1 µL each primer (10 pmol), 6 µL diethyl pyrocarbonate (DEPC)‐treated water and 2 µL cDNA. Mix was incubated for 10 min at 95°C by 1 cycle followed by 40 cycles including a denaturation step for 20 seconds at 95°C and a combined annealing step for 15 seconds at 72°C. Final extension was incubated for 10 minutes at 72°C. Amplification was done in 96‐well plates. Each plate consisted of cDNA samples and multiple water blanks as well as positive and negative controls. Separate amplification assays were done for *A2M*, alkaline phosphatase (*ALPL*), osteocalcin or bone gamma‐carboxyglutamic acid‐containing protein (*BGLAP*). Glyceraldehyde‐3‐phosphate dehydrogenase (*GAPDH*) as an internal control was used to normalize transcript levels of the current genes, and the results were analysed by the relative quantification 2^−ΔΔCt^ method. The primer sequences used in this study are listed in Table 1.

**Table 1:** Real‐time PCR primers (human)

Gene	Primer sequences	Product size (bp)
A2M	F: GAAGGAACAGTGGTGGAATTGAC R: CCGTGGTAGCATTGGAGTAATAG	214 bp
ALPL	F: CATGCTGAGTGACACAGACAAGAAG R: TGGTAGTTGTTGTGAGCATAGTCCA	126 bp
BGLAP	F: CCTCACACTCCTCGCCCTAT R: TGCTTGGACACAAAGGCTGC	111 bp
GAPDH	F: ATGTTCGTCATGGGTGTGAAC R: CACAGTCTTCTGGGTGGCAG	178 bp

Abbreviations: A2M, alpha‐2‐macroglobulin; ALPL, alkaline phosphatase; BGLAP, bone gamma‐carboxyglutamic protein; GAPDH; glyceraldehyde 3‐phosphate dehydrogenase.

### 2.13 Statistical analysis

Relative expressions were quantified using the relative Cq method with *GAPDH* as a reference gene. Data were represented as the Mean ± SD. To compare the outcomes, the paired sample *t* test was performed, and *P*‐values < .05 were regarded statistically significant.

## 3 RESULTS

Identification of MSCs from other mononuclear cells was revealed by their morphology and phenotype as well as their potency to differentiate to multilineage as described later. The cells formed colonies after 28 days of expanding.

### 3.1 Surface markers of MSCs

**Figure 1:**
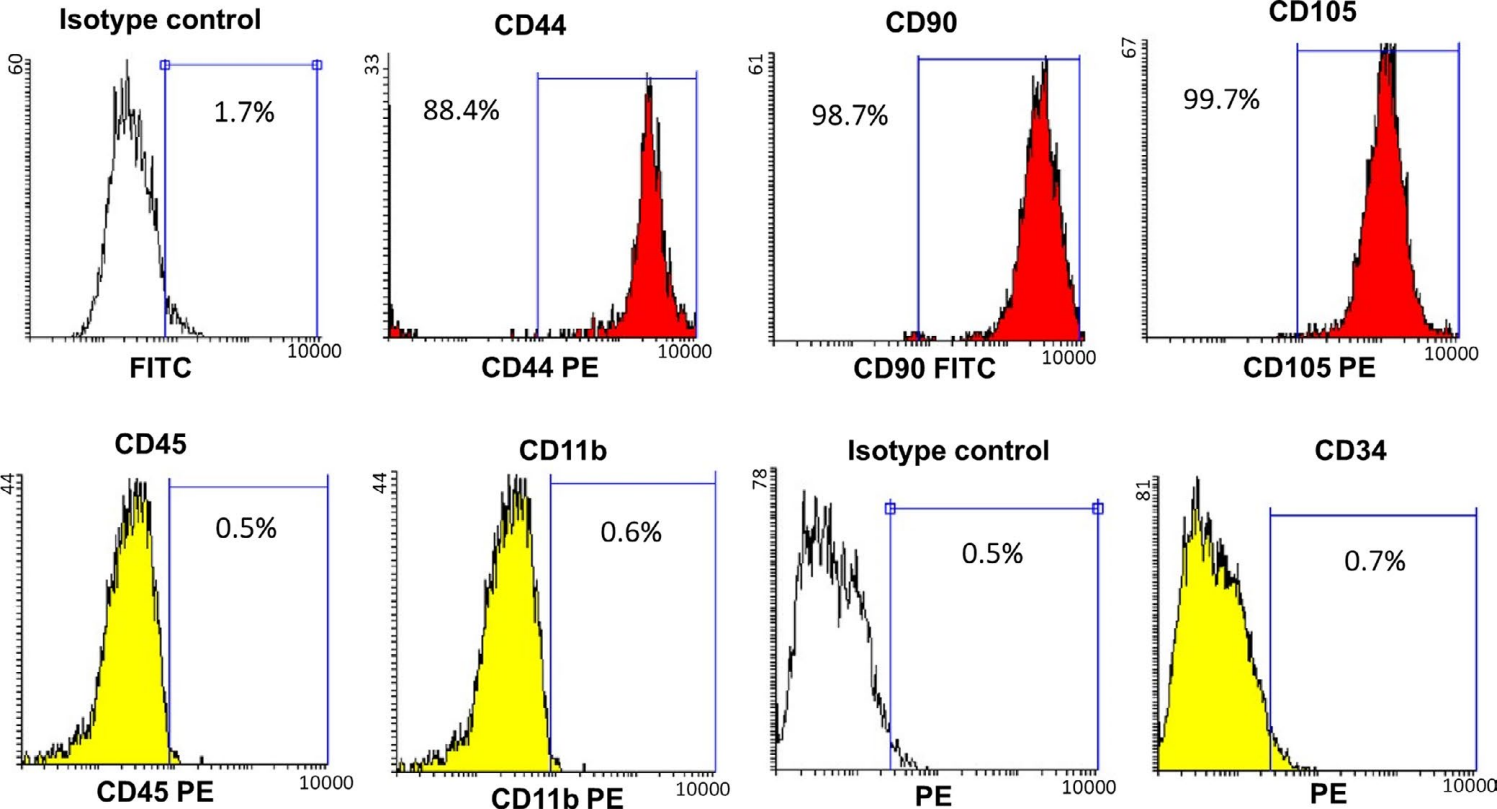
MSCs characterization using flow cytometry. The absence of hematopoietic lineage markers CD45, CD34 and CD11b and presence the cell surface markers of MSCs

The immunophenotype features of MSCs were analysed by flow cytometry. The results shown in Figure 1 revealed that MSCs were positive for CD44, CD90 and CD105; however, they were negative for CD45, CD34 and CD11b.

### 3.2 Multilineage differentiation of MSCs

**Figure 2:**
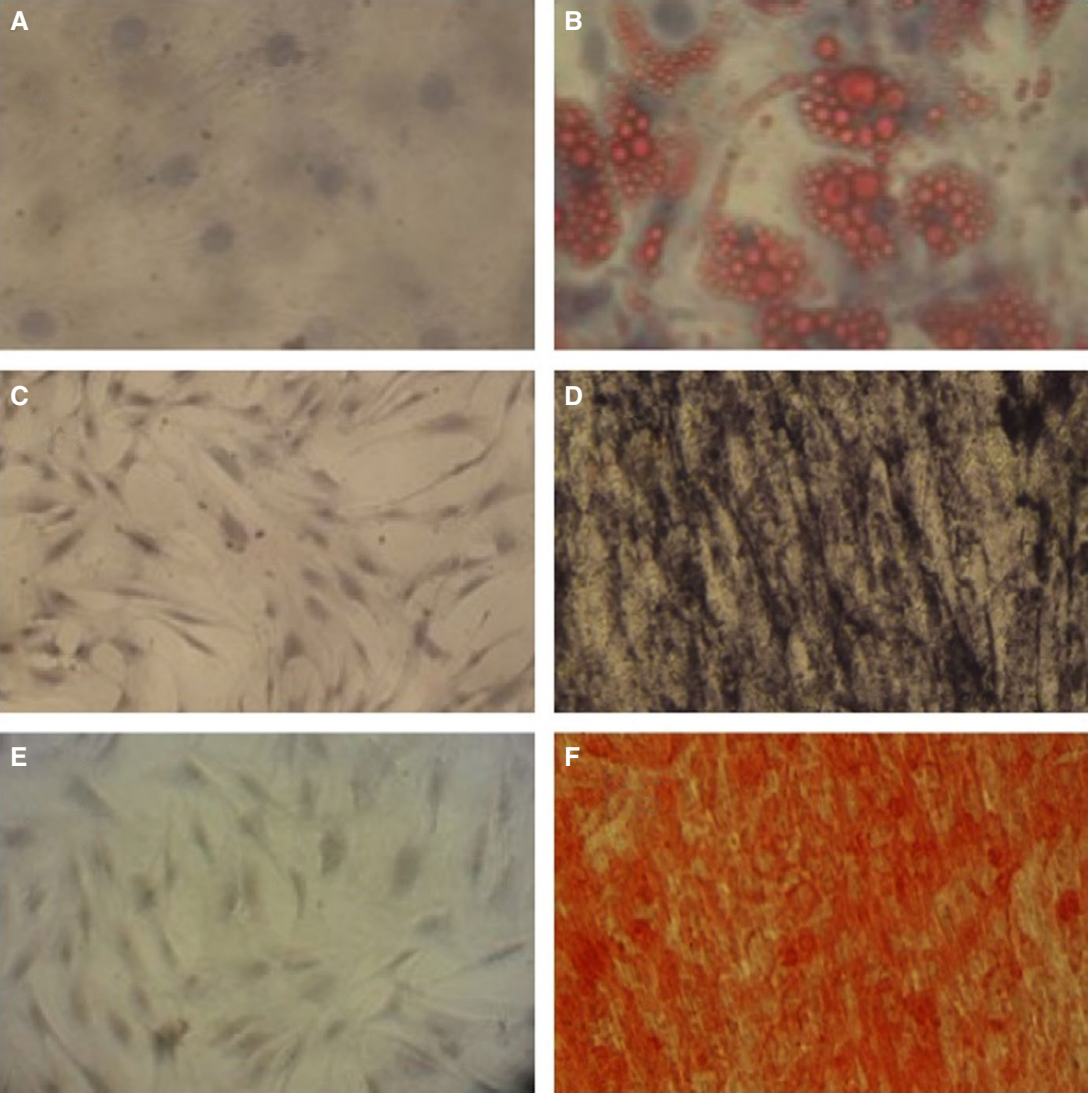
Multipotential differentiation of MSCs‐adipocyte staining: Control group (A) Differentiation to adipocyte was demonstrated by Oil Red O staining, (B)—Osteoblast staining: Control group (C) and (E), osteoblast differentiation was detected by alkaline phosphatase (D) and Alizarin Red S (F) staining

The hBM‐MSCs were differentiated to various cell types to show their pluripotency. So, hBM‐MSCs‐ were cultured for about 2 and 3 weeks in adipogenic and osteogenic differentiation medium, respectively. Oil Red O staining revealed droplets of fat in adipocytes (Figure 2B). Alkaline phosphatase activity was detected in differentiated MSCs (Figure 2D). Additionally, Alizarin Red S staining showed the calcium‐rich deposits in red (Figure 2F).

### 3.3 Gene expression analysis

**Figure 3:**
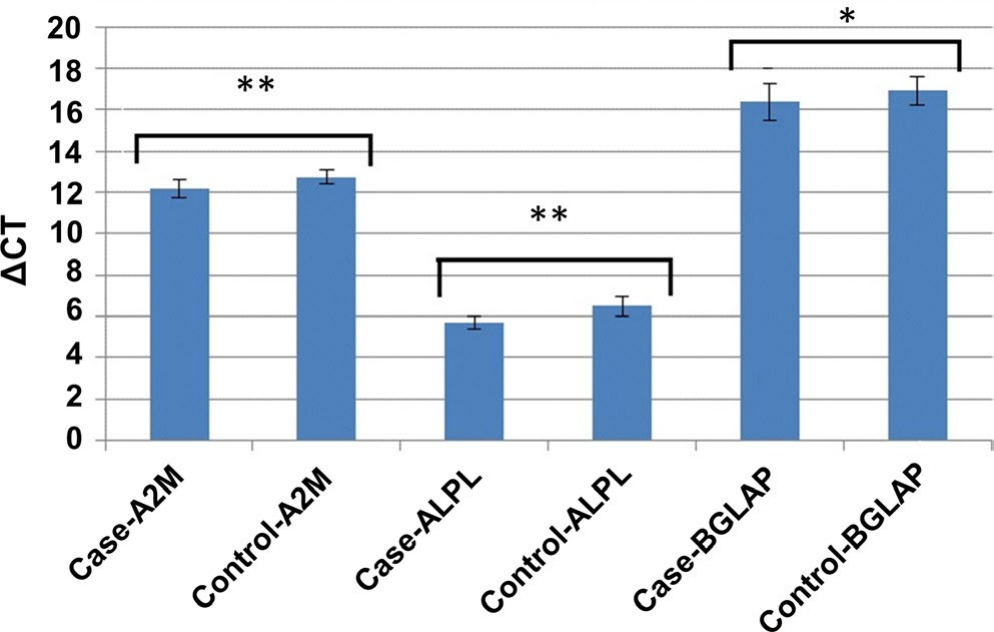
ACTH increases ALPL (fold change: 1.85), BGLAP (fold change: 1.56), A2M (fold change: 1.56), expression. Statistic: paired sample *t* test: *P < .05, **P < .01

After induction of MSCs into osteoblasts, ACTH significantly up‐regulated the expression levels of genes regarding to osteoblast maturation and function, including *ALPL* and osteocalcin (*BGLAP*). This hormone also significantly increased *A2M* expression (Figure 3). Besides, differentiated osteoblasts exposed to ACTH revealed a significant difference at transcriptional levels of *A2M*, *ALPL* and *BGLAP* in comparison with control groups, indicating a positive role of ACTH on enhancing transcriptional level of *A2M* as well as osteoblast markers.

## 4 DISCUSSION

Since MSCs have a great impact on bone regeneration, in the present study hBM‐MSCs were isolated and characterized. Results strongly pointed that isolated hBM‐MSCs revealed MSCs properties, since they formed colony and differentiated to osteogenic and adipogenic lineages.

It was shown that in vitro MSCs could attach to the plastic surface and generate colonies, and thus, they were considered as fibroblastoid colony‐forming cells.^18^ In vivo, MSCs at an injured bone site could proliferate and differentiate into osteoblasts.^19^ Hence, they could play crucial roles in repair of bone's fracture and injury. Previous investigations demonstrated that MSCs and osteoblasts express functional ACTH receptor (MC2R), and ACTH could affect the bone cells outside of hypothalamic‐pituitary‐adrenal axis.^20^, ^21^ This hormone could enhance osteoblastic differentiation of MSCs.^6^ To better understand the effect of ACTH on differentiated osteoblasts, the gene expression outlines and the related pathways were studied in the current study. Gene expression analysis demonstrated the enrichment of osteoblast markers (*BGLAP* and *ALPL*), which reveals the osteogenic enhancer effect of ACTH. It is interesting to mention that there was no significant difference between 10^−7^‐10^−12^ mol/L concentrations of ACTH. So, the authors selected 10^−8^ mol/L concentration based on their own experiments and other reports.^6^, ^22^ This hormone increased *A2M* expression significantly during hBM‐MSCs differentiation to osteoblasts and also in differentiated osteoblasts.

The femoral head has a very high rate of bone formation and resorption.^23^ The leading aetiology in destruction of femoral head called ‘ANFH’ is apoptosis of femoral head's bone. It has been shown that patients suffering from ANFH have obstructed blood vessels.^9^, ^24^ Reduction in shear stress due to declined blood flow could cause apoptosis of endothelial cells, which can eventually contribute to plaque erosion and thrombus formation.^25^ Several genes such as *A2M*, which contributes to coagulation and osteogenesis, could play a role in the pathogenesis of ANFH.^10^

**Figure 4:**
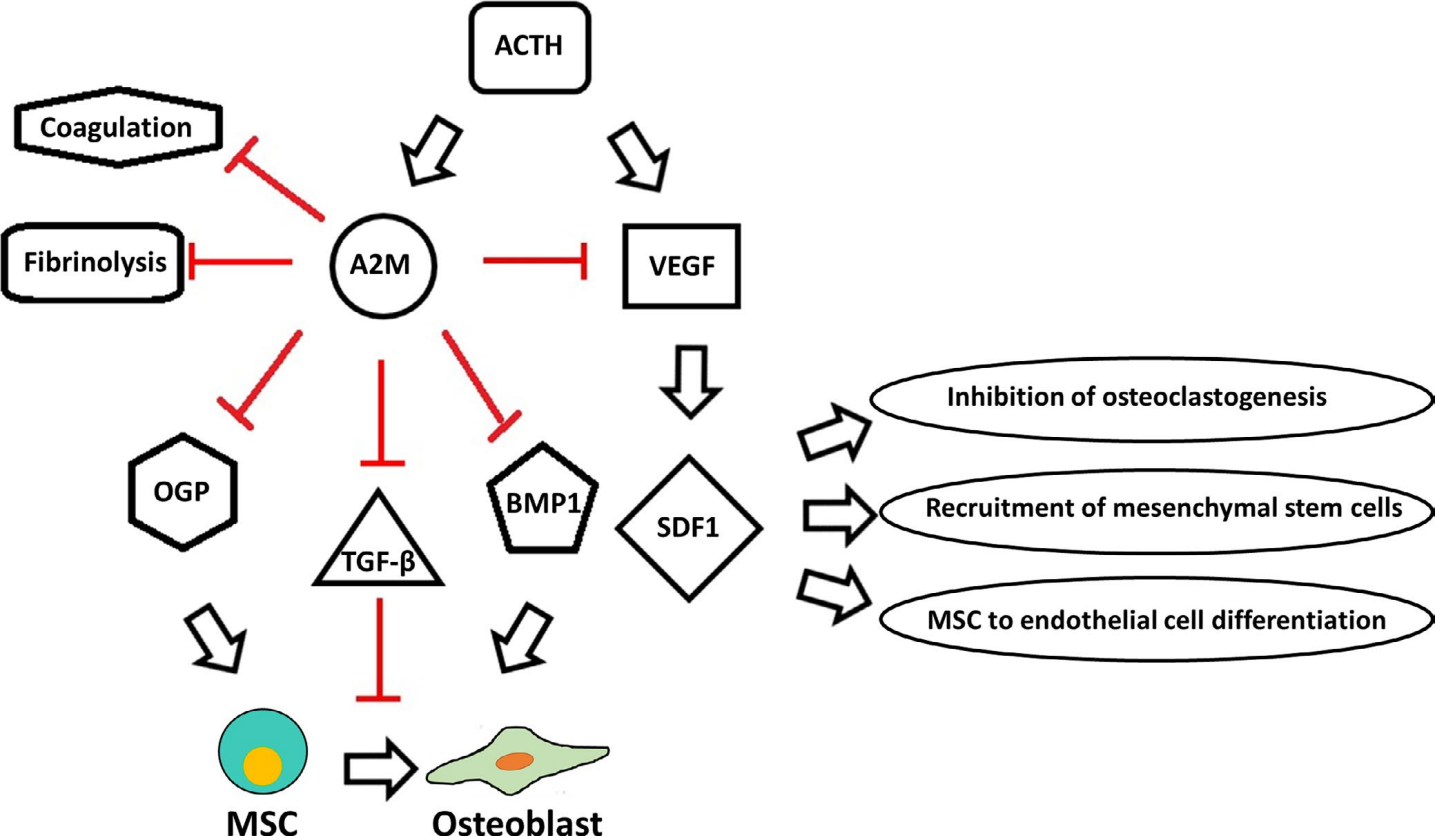
ACTH effects on osteogenesis, coagulation and fibrinolysis through A2M action

*A2M* is an anti‐protease that inactivates large variety of proteinase such as thrombin, contributing to coagulation, and plasmin as well as kallikrein contributing to fibrinolysis pathways.^12^, ^26^ It is able to bind to numerous growth factors and cytokines such as bone morphogenetic protein 1 (*BMP‐1*), transforming growth factor beta (*TGF‐β*), osteogenic growth peptide (*OGP*) and vascular endothelial growth factor (*VEGF*).^13^, ^16^ All these proteins play significant roles in osteogenesis, except *TGF‐β*,^27^ which promotes osteoblastic differentiation in the early stage and inhibits the late stage of osteogenesis.^27^ It is worth noting that *A2M* suppresses the function of *VEGF* and *BMP‐1*.^14^, ^16^ Thus, it seems that *A2M* has a dual effect in osteogenesis process. On the one hand, it affects *VEGF* and *BMP‐1,* but on the other hand it has an effect on *TGF‐β* (Figure 4).

ACTH, as an osteoblastic differentiation enhancer, up‐regulates *A2M*, which promotes osteoblastic differentiation probably through *TGF‐β* induction. Still, many topics need further investigations about the inhibiting effects of *A2M*, such as defining temporal order of activation pathways, studying pathway interaction, investigating the final effect of TGF‐β on bone formation, assessing intensity ranking of pathways and studying the differentiation phase of *A2M* up‐regulation.

## 5 CONCLUSION

In summary, the results have proposed a bilateral role of ACTH as along with *A2M* in bone regeneration. Further investigation is required to clarify the profile expression of this marker to explain the exact role of it in ANFH pathogenesis.

## CONFLICT OF INTERESTS

The authors declare no conflict of interest with respect to this research.

## AUTHORS’ CONTRIBUTIONS

MAK supervised the study. MAK and HNM participated in study design and scientific discussion of the data. FS and EV contributed to performing the experiments.

## CONSENT FOR PUBLICATION

All authors read and approved the final manuscript.

## Data Availability

The data that support the findings of the present study are available from the corresponding author upon reasonable request.
